# Plant Lectins and Lectin Receptor-Like Kinases: How Do They Sense the Outside?

**DOI:** 10.3390/ijms18061164

**Published:** 2017-05-31

**Authors:** Kevin Bellande, Jean-Jacques Bono, Bruno Savelli, Elisabeth Jamet, Hervé Canut

**Affiliations:** 1 Laboratoire de Recherche en Sciences Végétales, Université de Toulouse, CNRS, UPS, 24 Chemin de Borde Rouge, Auzeville, BP 42617, 31326 Castanet-Tolosan, France; kevin.bellande@lrsv.ups-tlse.fr (K.B.); savelli@lrsv.ups-tlse.fr (B.S.); jamet@lrsv.ups-tlse.fr (E.J.); 2 LIPM, Université de Toulouse, INRA, CNRS, 31326 Castanet-Tolosan, France; Jean-Jacques.Bono@toulouse.inra.fr

**Keywords:** lectin, lectin receptor-like kinase, carbohydrate, cell wall, signalling, plant development, plant pathology

## Abstract

Lectins are fundamental to plant life and have important roles in cell-to-cell communication; development and defence strategies. At the cell surface; lectins are present both as soluble proteins (LecPs) and as chimeric proteins: lectins are then the extracellular domains of receptor-like kinases (LecRLKs) and receptor-like proteins (LecRLPs). In this review; we first describe the domain architectures of proteins harbouring G-type; L-type; LysM and malectin carbohydrate-binding domains. We then focus on the functions of LecPs; LecRLKs and LecRLPs referring to the biological processes they are involved in and to the ligands they recognize. Together; LecPs; LecRLKs and LecRLPs constitute versatile recognition systems at the cell surface contributing to the detection of symbionts and pathogens; and/or involved in monitoring of the cell wall structure and cell growth.

## 1. Introduction

Lectins are widespread proteins in all kingdoms of life and have been identified as carbohydrate-binding proteins for a long time [1]. Their capability to selectively recognize and bind reversibly to specific mono- or oligosaccharides is the main feature of these proteins. Also, to be defined as lectins, carbohydrate-binding proteins have to display no enzymatic activity towards the recognized sugars and should not belong to the immunoglobulin family. Consequently, lectins group highly diverse protein families with considerable structure diversity as revealed by their folds and carbohydrate-binding site architectures [2]. They are soluble or membrane proteins, present both inside and outside cells. Carbohydrates, the potential ligands of lectins, belong to a large group of molecules with enormous diversity and complexity in structures. They also exist at the cell surface or inside cells. In the extracellular matrix, they form large complex networks of polysaccharides and proteoglycans. In plants, polysaccharides are the principal components of the cell wall [3,4]. In animals, proteoglycans possess glycosaminoglycans which are unbranched polysaccharide chains of 70 to 200 sugar residue-long, covalently linked to a core protein [5]. In both plants and animals, carbohydrates are also present to a great extent in glycoconjugates such as glycoproteins or glycolipids [6,7,8].

To cope with the complexity of carbohydrates, lectins exhibit varied molecular mechanisms allowing diversity and selectivity for sugar recognition. The binding sites of lectins contain polar residues that form hydrogen bonds with the highly abundant hydroxyl groups of sugars. Therefore, extensive networks of hydrogen-bonding arise and play a key role in ligand specificity. Aromatic side chains of tryptophan, tyrosine and phenylalanine residues also play critical roles by stacking the hydrophobic face of sugars. Calcium or magnesium ions in the binding sites of lectins mediate direct co-ordination bonds with the sugar itself [2,9,10,11,12,13]. These mechanisms are shared within lectin families to bind a core monosaccharide at a primary binding site, but high selectivity and diversity are achieved through extended and secondary binding sites that strengthen contacts with oligosaccharides or glycoconjugates. The extension of binding sites is often unique to individual members of the family [10]. Even in combination, these bonds only provide a weak affinity to the ligand in the millimolar range. However, a strong affinity (micromolar to nanomolar range) is often obtained with multivalent interactions. Both carbohydrate-binding sites and ligands can be multivalent thanks to the branched structures of glycans or to the clustering of cell surface glycans or polysaccharides. Clustered carbohydrate-binding sites can result either from the association of two or more lectin domains in a single protein, or from oligomerisation of monovalent proteins [9]. Di- and tetramerisations are well described for lectins. Even more, oligomerisation generates several mutual orientations of the subunits leading to a variety of quaternary structures with implications in ligand recognition [14]. Finally, members of all the lectin families are widely distributed among the life kingdoms and each organism manages a complex panel of lectins [14]. It suggests a role for lectins in deciphering the biological information contained in carbohydrates.

In plants, lectins have been early described as important players in defence against pathogens [15]. Most of them are secretory proteins routed either to the vacuole where they accumulate in large amounts in seeds, or to the cell wall and the plasma membrane. The structures of the members of the different plant lectin families are extensively described, but their function is somewhat puzzling [1,2]. The wealth of information provided by genomic sequencing of various plant species, transcriptomics and proteomics studies, as well as reverse genetics approaches, has been invaluable to describe the full complement of lectins in many plant species, and to understand their functions. New families of lectins are emerging, e.g., lectins that are synthesized in the cytoplasm and reside in the cytoplasmic/nuclear compartments. Their location in the nucleus may have important impacts and questions the nature of their potential ligands and their physiological roles [16,17]. The sequencing of plant genomes also reveals a great deal of chimerolectins, i.e., a single protein consisting in a lectin domain associated to an unrelated domain [1]. For example, in *Arabidopsis thaliana* (*A. thaliana*), Legume-type lectins (L-type) are associated with intracellular kinase domains to constitute receptor-like kinases (LecRLKs) [18,19,20], thus focusing research on the roles of lectins in signalling. Besides the classical lectins, polysaccharide recognition is also achieved by carbohydrate-binding modules (CBMs). Initially found in bacteria and fungi, CBMs are non-catalytic domains frequently appended to glycoside hydrolases that degrade plant cell wall polysaccharides. Thereafter, they have been identified in all life kingdoms. They are grouped in families based on amino acid sequences and structures (available online: www.cazy.org) [21]. Noteworthy, CBMs bind to their ligands through mechanisms similar to lectins such as hydrogen bonding, hydrophobic interactions and Ca^2+^ coordination bonding. CBMs and lectins may have similar folds: the dominant fold among CBMs is the β-sandwich which is shared with the plant L-type lectins and the animal galectins [9,22]. In plants, CBMs can be appended to intracellular kinase and trans-membrane domains to constitute receptor-like kinases, thus assigning CBMs as for classical lectins a role in signalling [23,24].

In this review, we first describe the architectures of proteins harbouring C-type, G-type, L-type lectins, and LysM or malectin CBMs, and their distribution through kingdoms. The classification of lectins is based on their amino acid sequences, structures and properties: C-type lectins require calcium ions for carbohydrate binding; G-type lectins are *Galanthus nivalis* agglutinin-related lectins; L-type lectins have been identified in abundance in Legume seeds and LysMs in bacterial autolysins; malectin CBMs show binding to glucose oligomers (maltose). Second, we draw up an inventory of the *A. thaliana* and *Medicago truncatula* (*M. truncatula*) genes together with phylogenic trees derived from amino acid sequence alignments of lectin domains as defined in the Pfam and InterPro databases [25,26]. Sub-cellular location and post-translational modifications (PTMs) are analyzed by exploring proteomic data from public repositories. We then focus on the functions of lectin receptor-like kinases (LecRLKs) referring to the biological processes they are involved in and to the ligands they recognize, mainly using examples from the model plant *A. thaliana* and *M. truncatula*. The latter have been selected owing to the well documented functions of their LecRLKs in different aspects of the plant biology. We discuss the roles of the different protein architectures in the recognition of self and non-self molecules to provide adapted responses to controlled cell growth and environmental cues.

## 2. Occurrence of Lectin Domains in Plants

### 2.1. Distribution of Lectin Domains through Life Kingdoms

**Figure 1:**
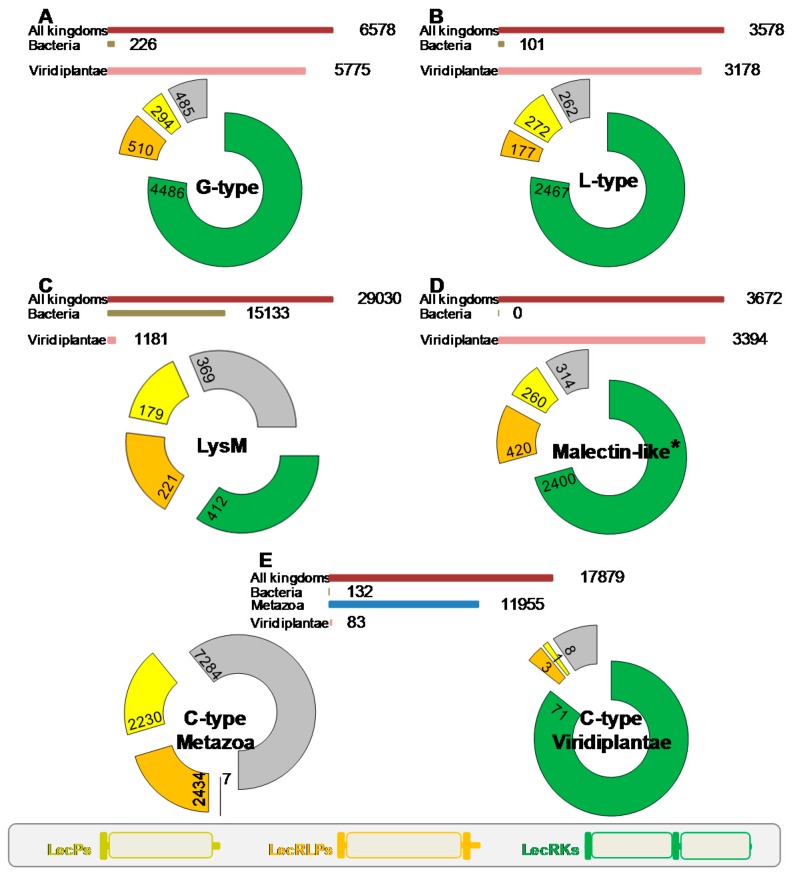
Distribution of lectin domains across kingdoms and ratio of domain organizations in Viridiplantae. Data were retrieved from Pfam (pfam.xfam.org; version 31, March 2017): the number of sequences including a predicted lectin domain is indicated in Viridiplantae (pink bar), in Bacteria (brown bar), and in all kingdoms (dark pink bar). The donut charts categorized in ratio different domain organizations (see inset, bottom of the figure): a lectin domain is associated with a kinase domain (green), with a signal peptide and a trans-membrane domain (orange), with a signal peptide only (yellow), others (grey). The number of sequences in each case is indicated as well. (**A**) G-type lectins; (**B**) L-type lectins; (**C**) LysM domains; (**D**) Malectin domains. * The family considered here is Malectin_like (PF12819) that is found in a number of receptor kinases. The Malectin family (PF11721) comprised 2349 sequences with the following distribution: Viridiplantae 1493, Metazoa 227, Bacteria 314; (**E**) C-type lectins, distribution is shown both for Metazoa (blue bar) and Viridiplantae organisms.

A first survey of the literature indicates that proteins having the architecture of LecRLKs belong to several lectin and CBM families, namely the G-type and L-type lectins [1], together with the LysM and malectin CBMs [23,24]. In order to address an overview of the domain organization of lectins among the different families, we carried out a comprehensive search using the Pfam database [25] (pfam.xfam.org; version 31, March 2017). The data mining concerned the above lectin families, but also all the CBM families [21] (available online: www.cazy.org) and the C-type lectin and jacalin families. We summarize the results of the search in Figure 1. First, lectins are widespread proteins and several thousand sequences were retrieved for each family. However, their expansion tremendously varied depending on the life kingdom. While G-type and L-type lectin families found large spreading in plants, LysM CBMs and C-type lectins are expanded in bacteria and in animals respectively. Malectin CBMs are referenced in two accessions, i.e., malectin domains (PF11721) and malectin-like domains (PF12819): both found large spreading in plants (Figure 1). Second, it appears that three domain organizations are predominant in most families: (i) the typical LecRLK architecture, i.e., from N- to C-terminus, signal peptide, lectin/CBM domain, transmembrane domain and intracellular kinase domain; (ii) the receptor-like protein (LecRLP) architecture, i.e., the same as previous except the lack of the kinase domain; (iii) the soluble protein (LecP) architecture, with only signal peptide and lectin/CBM domain.

The three lectin gene families that found large expansion in plants roughly present the same proportions of LecRLK, LecRLP and LecP architectures with a predominance of the LecRLK architecture (three fourths of the sequences) whereas LecRLPs and LecPs only account for 5–10% (Figure 1). Noteworthy, regarding the L-type lectins and the malectin CBMs, the LecRLK architecture was found neither in bacteria nor in animals. In the G-type lectin family, only three bacterial sequences show the LecRLK architecture. The LysM CBMs mainly found in bacteria are present in plants with 1181 sequences. One third of these proteins contain a kinase domain. LecRLPs and LecPs account for 20% each. Two sequences with the LecRLK architecture are found in bacteria (A0A0N0UXM0 and A1ZLP4, available online: www.uniprot.org) and none in animals. Surprisingly, among the twelve thousand sequences retrieved for C-type lectins in animals, only seven show the LecRLK architecture: LecRLPs and LecPs account for 20% each. Moreover, from the 83 sequences originated from plants, 71 present the LecRLK architecture. No LecRLK containing the C-type lectin domain is found in bacteria. Therefore, the LecRLK architecture essentially appears as a specificity of the plant kingdom.

A few lectin and CBM families include chimerolectins with a kinase domain. CBM 13 and 51 families comprise 26 and 14 LecRLKs respectively, all in bacteria. One can note two LecRLKs unique to plants in the genome of the liverwort *Marchantia polymorpha*: a kinase domain is appended to one or four CBM 18 defined as chitin binding modules [21] (Uniprot entries A0A176WS68 and A0A176WDS5 respectively). The jacalin family with a large expansion in plants reveals 26 sequences including a kinase domain. However, most of them are predicted to be cytoplasmic proteins [1]. It is also worthy of note 91 sequences from plants that consist in a predicted extracellular domain belonging to the GH 18 family (chitinases) and an intracellular kinase domain. The tobacco CHRK1 is one example of them and is assumed to play a role in plant development [27,28]. However, it cannot be considered as a LecRLK since the extracellular domain is predicted to have an enzymatic activity towards carbohydrates.

An inventory of the *A. thaliana* lectin genes is given in Table S1 including the C-type, G-type and L-type lectin families as well as the LysM and malectin CBMs. The proteins of *M. truncatula* that harbour a LysM CBM are listed in Table S2. Repertoires of LecRLKs are also available for poplar, rice and bread wheat [29,30,31]. In the main, lectin families that expanded in the plant kingdom are all present in *A. thaliana*. The LecRLK architecture is predominant and the occurrence of LecPs and LecRLPs in *A. thaliana* appears in similar proportions as in the plant kingdom. For instance, the L-type lectin family comprises 54 members with 43 LecRLKs (80%), seven LecPs (13%) and four LecRLPs (7%). The C-type lectin family has only one member. The LysM CBMs give a more complex picture with three predicted cytoplasmic proteins, five LecRLKs, five LecPs and the absence of LecRLPs. This is confirmed in *M. truncatula* with only one LecRLP (Table S2).

**Figure 2:**
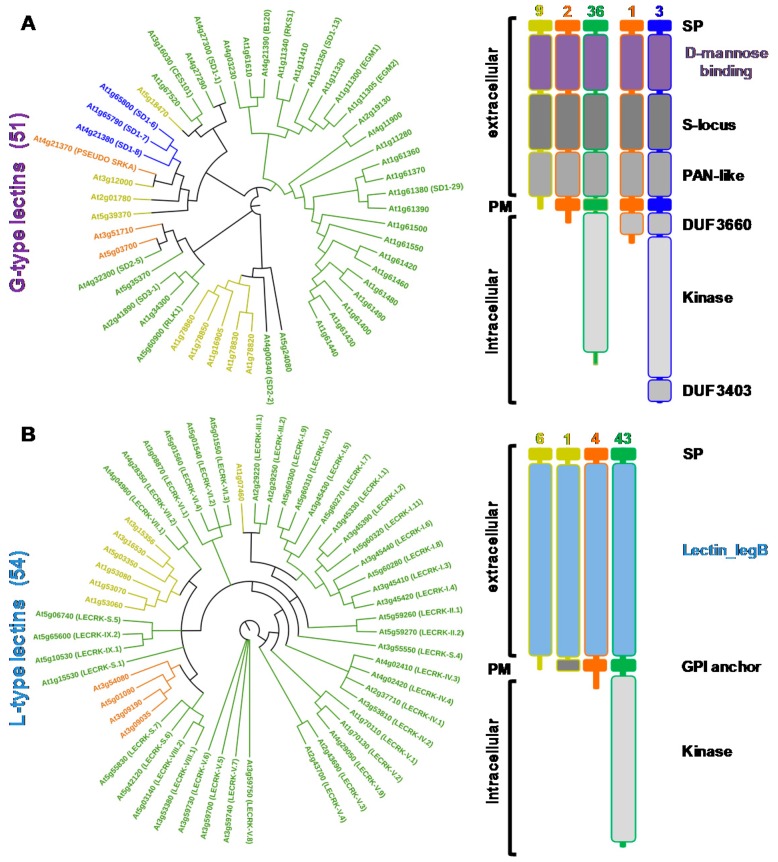
Phylogenetic analysis and domain organizations of *Arabidopsis thaliana* G-type and L-type lectins. Amino acid sequences of lectin domains as defined in the Pfam database (pfam.xfam.org) were analyzed using the Maximum Likelihood method based on the JTT (Jones, Taylor, Thornton) matrix-based model [34]. The bootstrap consensus tree was inferred from 1000 replicates and evolutionary analyses were conducted in Molecular Evolutionary Genetics Analysis (MEGA7.0) [35]. The display and annotation of the tree have been done with interactive Tree Of Life (iTOL) [36]. Branches with less 50% support were collapsed. The colour code refers to the protein architectures: LecPs (yellow), LecRLPs (orange), and LecRLKs (either green or blue) (Table S1). (**A**) G-type lectins: the extracellular moieties of proteins comprise a signal peptide (SP) followed by d-mannose binding domain, an *S*-locus glycoprotein domain, and a PAN-like domain [37]. Proteins with intracellular moieties harbour either a kinase domain alone (green) or a kinase domain appended to the domains of unknown function DUF 3660 and DUF 3403 (blue); (**B**) L-type lectins: the extracellular moieties of proteins only comprise a signal peptide and a Lectin_legB domain. Some of the LecPs have a predicted GPI (glycosyl phosphatidyl inositol) anchor. L-type LecRKs only comprise a kinase domain in the intracellular moiety. The number of LecPs, LecRLPs and LecRKs in the different domain organizations is indicated. PM: plasma membrane.

**Figure 3:**
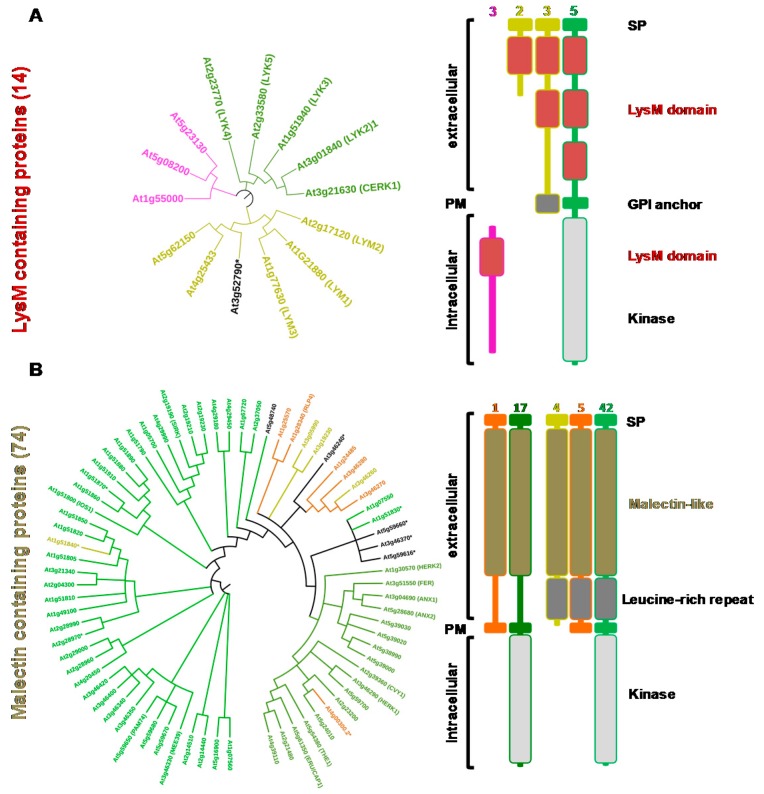
Phylogenetic analysis and domain organizations of *Arabidopsis thaliana* LysM and malectin CBMs. Amino acid sequences of CBM domains as defined in the Pfam database (pfam.xfam.org) were analyzed using the Maximum Likelihood method based on the JTT matrix-based model [34]. The bootstrap consensus tree was inferred from 1000 replicates and evolutionary analyses were conducted in MEGA7 [35]. The display and annotation of the tree have been done with iTOL [36]. Branches with less 50% support were collapsed. The colour code refers to the protein architectures: LecPs (yellow), LecRLPs (orange), LecRLKs (either green or dark green), cytoplasmic proteins (pink) and undetermined (black) (Table S1). (**A**) LysM containing proteins: the extracellular moieties of proteins only comprise a signal peptide (SP) and LysM domains. Some of the LecPs have a predicted GPI (glycosyl phosphatidyl inositol) anchor. LysM LecRKs only comprise a kinase domain in the intracellular moiety. The number of LecPs, LecRLPs and LecRKs in the different domain organizations is indicated; (**B**) Malectin containing proteins: the extracellular moieties harbour either a kinase domain alone (dark green) or a kinase domain appended to leucin-rich repeats (green). Malectin LecRKs only comprise a kinase domain in the intracellular moiety. PM: plasma membrane.

From the sequences of the lectin domains, phylogenetic trees were constructed and the domain organization of the proteins detailed for G-type and L-type lectins (Figure 2) as well as for LysM and malectin CBMs (Figure 3). C-type lectins were removed from the analysis because of their scarcity in plants. Interestingly, for each family, the LecRLK, LecRLP and LecP architectures segregate in different groups thus forming distinct clades. This result has been previously observed for the L-type lectins [32] indicating that LecPs and LecRLPs share independent evolutionary histories with LecRLKs. The observation is here extended to the other lectin families. However, the phylogenetic tree for the G-type lectins shows one clade of seven members that groups the three architectures (Figure 2A). The intracellular moieties of the three LecRLKs and the LecRLP present in the clade reveal the presence of additional domains, namely DUF 3660 and DUF 3403. It suggests a distinct evolution for the proteins present in the clade. Two of them are located at the *S*-locus of *A. thaliana* and are involved in self-compatibility (see Section 3) [33]. As mentioned above for L-type lectins, LecRLPs and LecPs group in distinct clades, except At1g07460 that comes as a singleton (Figure 2B).

At1g07460 is the only L-type LecP for which a GPI (glycosyl phosphatidyl inositol)-anchor is predicted (Table S1). Similarly, the LysM containing LecPs segregate in a clade: one branch groups three proteins with a predicted GPI anchor, namely LYM 1, 2, and 3 (Figure 3A). It should be noted that LysM-containing proteins usually harbour three contiguous LysM domains, but some of them are poorly conserved and thus not predicted (Tables S1 and S2). The malectin-containing proteins mainly subdivide in two groups following domain organization, i.e., presence/absence of leucine-rich repeat (LRR) domains (Figure 3B). The two groups also differ by the exon-intron organization of the corresponding genes (Table S1). The proteins that harbour only the malectin domain in their extracellular moiety are 17 LecRLKs and one LecRLP. The corresponding genes display only one exon. These proteins are also named *Catharanthus roseus* (*C. roseus*) RLK1-like (CrRLK1L), after the first member has been identified in *C. roseus* cell cultures [24]. The second group contains 42 LecRLKs, five LecRLPs and four LecPs. They have been considered as LRR receptor-like kinases (LRR I subfamily) [38]. Therefore, *A. thaliana* deals with a large complement of lectins (close to 200 proteins) with differences in protein architecture.

After signal peptide, GPI-anchor and transmembrane domain predictions, LecRKs, LecRLPs and LecPs are assumed to be located either in the vacuole, in the cell wall or at the plasma membrane. In the following paragraph, we look at proteomics data to discuss their sub-cellular location and post-translational modifications.

### 2.2. LecRKs, LecRLPs and LecPs Are Located at the Cell Surface and Exhibit PTMs: A Proteomics View

In the recent years, proteomics has expanded our knowledge of the protein complement of plant organs and organelles thanks to progresses in mass spectrometry technologies, genome sequencing and bioinformatics. A lot of experimental data are available and they cover many model plants or crops as well as different sub-cellular compartments [39,40]. In this review, we are mainly interested in the plant cell surface. Hence, we have done a systematic search for the identification of LecRKs, LecRLPs and LecPs in cell wall, GPI-anchored and plasma membrane proteomes, as a mean to get information about the sub-cellular location of these proteins (Tables S3 and S4). In addition, we have collected in these proteomes the available data regarding their PTMs, i.e., phosphorylation, *N*-glycosylation and GPI-anchor.

LecPs of all the described families (G-type, L-type, malectin and LysM) are found in *A. thaliana* cell wall proteomes of different organs and of cell suspension cultures (Table S3). About half of the expected LecPs are presently identified (five G-type, five L-type, one malectin, three LysM). This proportion is consistent with the present size of the known *A. thaliana* cell wall proteome which comprises nearly 900 proteins, i.e., about one half of the expected one (see www.polebio.lrsv.ups-tlse.fr/WallProtDB/—March 2017). LecPs have been identified in other dicots (*Brassica oleracea*, *Medicago sativa*, *Linum usitatissimum*, *Gossypium hirsutum* and *Solanum lycopersicum*) as well as in monocots (*Brachypodium distachyon* and *Oryza sativa*). Remarkably, only three LecPs have been identified out of 189 proteins in the xylem sap of *Brassica oleracea* [41] and only one out of 30 in that of *Gossypium hirsutum* [42]. The presence of these LecPs in extracellular proteomes is consistent with the bioinformatic prediction of their localisation. In addition, several of them are found in *N*-glycoproteomes obtained after affinity chromatography of proteins on Concanavalin-A (ConA) sepharose which is based on the recognition of *N*-glycans by ConA [43,44,45,46,47]. Several LecPs are also identified in GPI-anchored proteomes prepared from purified plasma membranes from which proteins are released by phospholipase C or D [48,49,50,51].

In addition to these LecPs predicted to be either soluble or GPI-anchored, several LecRKs and one LecRLP are found in these proteomes. In all cases, the identified peptides are located in the extracellular domains of the proteins. Most of them are found in either a *N*-glycoproteome [43,45,47] or a GPI-anchored proteome [51].

Then, the search for LecRKs and LecRLPs has been focused on the thirteen available plasma membrane proteomes of *A. thaliana* prepared from roots, seedlings, leaves, inflorescences and cell suspension cultures (Table S4): 83 LecRKs (21 G-type, 23 L-type, five LysM, 42 malectin) and six LecRLPs (two G-type, four malectin) have been identified. The coverage of the whole family is of about fifty percent. Among these proteins, 38 are identified in a phosphoproteome [52] and phosphorylation sites could be located in the kinase domain for 21 of them [53,54,55]. Interestingly, several LecPs have been also identified in these plasma membrane proteomes (two G-type, five LysM, one malectin).

The fact that the cell wall, GPI-anchored and plasma membrane proteomes share some proteins suggests the existence of interactions between them. Indeed, in each case, several steps of purification are performed to enrich the analyzed fractions in the proteins of interest. As expected, LysM LecPs are found in a GPI-anchored proteome, but some of them are also identified in a plasma membrane proteome. Unexpectedly, two G-type LecPs are found in a plasma membrane proteome and LecRKs with malectin domains are found in a GPI-anchored proteome. This observation is consistent with the identification of the *Capsicum annuum* G-type LecP, CaMBL1, in both a microsomal fraction and a soluble fraction including secreted proteins [56]. Besides, CaMBL1 was localized at the plasma membrane after transient expression of a CaMBL1-Green Fluorescent Protein (GFP) fusion protein in onion epidermal cells [56].

Thus, LecPs, LecRLPs and LecRLKs are readily found at the cell surface. LecRLKs have been shown to dimerize upon ligand recognition [57]. Given the facts that (i) di- or tetramerisation is a characteristic feature of lectins [2,14] and (ii) multivalency is critical for specificity of recognition and affinity, it is tempting to consider signalling complexes combining LecPs, LecRLPs and/or LecRLKs. In this way, ligand-induced homo-dimerisation and hetero-tetramerisation involving LecPs and LecRLKs find examples in the LysM family (see Section 5).

## 3. G-Type Lectin Receptor-Like Kinases

G-type LecRLKs of *A. thaliana* comprise a d-mannose binding lectin domain at the N-terminus of the protein followed by an *S*-locus glycoprotein domain and a PAN-like domain (Figure 2). In few members of the family, either the *S*-locus and/or the PAN-like domains are poorly conserved and then not predicted (Table S1). The lectin module of about 100 amino acid residues presents a β-trefoil fold consisting of a repeat of three subdomains of four-stranded β sheets, each of them harbouring a unique carbohydrate-binding site [1,2]. This module was initially considered as a mannose-specific lectin, but some of them exhibit strong affinity toward oligomannosides and high-mannose *N*-glycans [1]. The *S*-locus domain (about 100 residue-long) originates from *S*-locus glycoproteins, as well as from *S*-receptor kinases (SRKs) which play a role in self-incompatibility in the *Brassicaceae* family (see below). It might have an epidermal growth factor (EGF)-like structure and is sometimes reported as EGF-like domain [58]. The PAN-like domain (about 80 residue-long), as the *S*-locus domain, contains conserved cysteine residues involved in the formation of disulfide bridges. PAN modules fulfil diverse functions by mediating protein-protein or protein-carbohydrate interactions [37].

In *A. thaliana*, G-type LecRLPs and LecRLKs are involved in a variety of important biological processes, i.e., adaptation to environmental cues, pathogen detection and defence responses, as well as reproduction. For example, the *S*-domain receptor kinase1-6 (SD1-6/At1g65800) is implicated in regulating lateral root development under phosphate-starvation [59]. Two G-type LecRLKs, namely EGM1/At1g11300 and EGM2/At1g11305, are potentially involved in signalling of mannitol-associated stress responses [60]. Interestingly, the responses are not linked to the osmotic stress, but instead to a specific chemical property of mannitol itself. It is suggested that the genes could be activated by mannitol produced by pathogens and may contribute to plant defence. Together with SD1-29 (At1g61380), which mediates bacterial lipopolysaccharide sensing [61], several G-type LecPs and LecRLKs from *A. thaliana* and other plant species are involved in plant pathogen interactions [56,62,63,64,65,66,67,68]. Besides, the SRKs in the *Brassicaceae* family, which are G-type LecRLKs, focus abundant studies due to the importance of self-incompatibility in flowering plants (for reviews, see [58,69]).

Self incompatibility is a genetic barrier for self-pollen rejection. In the genus *Arabidopsis* and other genera of the *Brassicaceae* family, recognition specificity is achieved by the interaction of SRK located at the epidermal cells of stigma and its ligand SCR (*S*-LOCUS CYSTEINE-RICH PROTEIN) present in the coat of pollen grains. The receptor and the ligand genes are physically linked at the *S*-locus. However, most of *A. thaliana* cultivars are self-fertile. In particular, the *S*-locus of *A. thaliana* Columbia-0 (Col.-0) has been shown to contain non-functional SRK and SCR sequences [33]. The truncated SRK (PSEUDO-SRKA/At4g21370) is devoid of intracellular kinase and DUF3403 domains and has a LecRLP architecture (Figure 2). Since the transformation of *A. thaliana* Col0 or C24 with functional *SRK* and *SCR* gene pairs restores self-incompatibility [70,71], SRK and SCR have been defined as the primary determinants of self-incompatibility. The binding of SRK to SCR leading to pollen rejection occurs through protein–protein interactions and only if receptor and ligand are encoded by the same *S*-locus. Within the SRK receptor, the involved amino acids are located in two hypervariable regions: the first is about 20 amino acid-long and is located in the middle of the d-mannose binding domain; the second one is 30 amino acid-long and covers part of the d-mannose binding and the *S*-locus (EGF-like) domains [72]. It is believed that the variable residues in these regions act as self-incompatibility specificity determinants [72]. Therefore, the d-mannose binding domain of SRKs functions in protein-protein interactions. SRKs are highly *N*-glycosylated in stigmas with a few glycan moieties located in the extracellular ligand binding domain. While *N*-glycosylation is not important for SCR-dependent activation of SRK, it ensures a proper subcellular trafficking of SRK to the plasma membrane [73]. SRKs form dimers in the absence of ligand. Even more, the dimerisation seems to be a prerequisite to ligand binding since the monomeric form of SRKs cannot bind SCR [74,75]. The dimerisation does not require protein glycosylation and is mediated by the PAN-like domain.

Data about the signalling pathways underlying G-type LecRLK-dependent responses are restricted to SRKs. Two proteins, MLPK (*M*-LOCUS PROTEIN KINASE) and ARC1 (ARM-REPEAT CONTAINING 1), have been shown to interact with the kinase domain of SRKs and both are essential for the completion of self-incompatibility. MLPK is a membrane-associated kinase existing in two isoforms that differ in anchoring to the plasma membrane through either myristoylation or hydrophobic sequence [76]. ARC1 is a U-box/ARM repeat-containing protein that is phosphorylated by both SRK and MLPK [77]. ARC1 which is an E3 ubiquitin ligase can ubiquitinate multiple targets upon incompatible pollination [78]. Interestingly, in yeast two-hybrid analyses, various kinase domains of G-type LecRLKs from *A. thaliana* and *Brassica* have been shown to interact with representative U-box/ARM repeat domains of *A. thaliana* E3 ubiquitin ligases [79]. It suggests a conserved signalling pathway for G-type LecRLKs.

The G-type LecRLK At1g61380 (SD1-29) has been recently involved in the bacterial lipopolysaccharide (LPS) sensing [61]. LPS is a well described MAMP (microbe-associated molecular pattern) in mammalian cells with the lipid moiety responsible for much of the toxicity of Gram-negative bacteria. Similarly, in *A. thaliana*, the lipid moiety of LPS from *Pseudomonas* species is also sufficient to trigger elevations in cytoplasmic Ca^2+^ concentrations. SD1-29 was renamed LORE (LIPOOLIGOSACCHARIDE-SPECIFIC REDUCED ELICITATION) after its identification in a screen for LPS-insensitive mutants [61]. Noteworthy, the yeast two-hybrid analyses of various kinase domains of G-type LecRLKs with U-box/ARM repeat domains of E3 ubiquitin ligases have revealed LORE which could interact with all the ARM repeat domains tested [79]. It is tempting to speculate that E3 ubiquitin ligases are involved in pathways after LPS elicitation. However, it is not known if LORE binds to LPS through its oligosaccharide or lipid moiety. Presently, the solely described interaction involving a d-mannose binding domain associated to a LecRLK is the SRK/SCR pair through protein-protein interaction.

## 4. L-Type Lectin Receptor-Like Kinases

*A. thaliana* Legume-type LecRLKs exhibit a unique lectin module consisting of about 250 amino acid residues at the N-terminus of the protein. This module presents the typical β-sandwich fold of the legume lectins, which consists of a flattened six-stranded β-sheet (back face) and a curved seven-stranded β-sheet (front face) [2,18,80]. Both faces are interconnected by turns and loops forming a single potential carbohydrate-binding site. Two divalent cations Ca^2+^ and Mn^2+^ are incorporated in the structure, thus stabilizing the carbohydrate-binding conformation. No other domain is appended to the lectin module in the extracellular moiety as for LecPs and LecRLPs.

A comprehensive expression analysis of all the L-type *LecRLK* genes has been performed by exploring public repositories [81]. This revealed that most of the *LecRLK* genes are not or weakly expressed in all of the *A. thaliana* tissues and developmental stages, with the notable exception of three of them, namely *LecRK-I.9*, *LecRK-IV.1*, and *LecRK-VIII.1* the transcripts of which are found in all tissues. In contrast, the expression of many genes changes in response to various stimuli: hormones, abiotic stress, but also a variety of elicitors. Consistent with this observation, strong gene induction and repression have been measured upon interaction with pathogens. The idea that L-type LecRLKs play important roles in plant defence is supported by experimental studies, some of them based on the study of knock-out mutants. *LecRLK* genes are then involved in plant-insect [82], -bacteria [83,84,85,86], -oomycetes [87,88,89] and -fungi interactions [90]. In addition, a systematic screen of *A. thaliana LecRLK* T-DNA insertion lines identified several additional LecRLKs that are involved in resistance against either *Alternaria brassicicola*, Phytophthora or Pseudomonas pathogens [91]. On the other hand, only few genes are involved in developmental processes [92,93].

**Figure 4:**
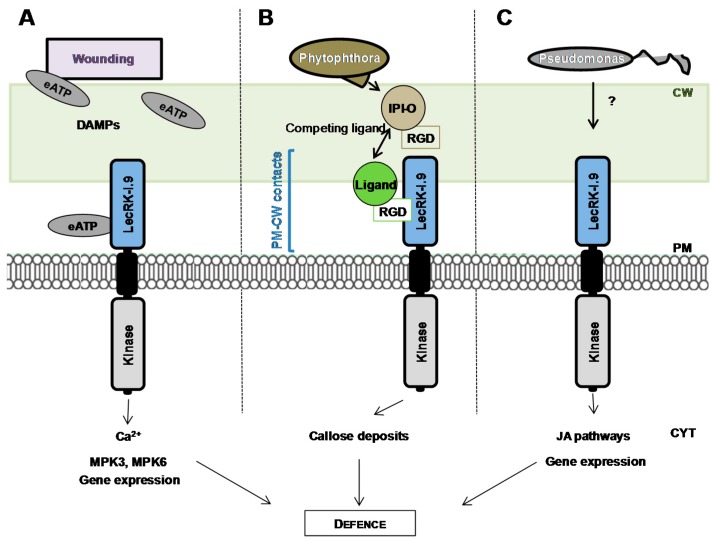
*A. thaliana* L-type lectin receptor kinase LecRK-I.9 and its putative ligands. (**A**) Extracellular ATP (eATP) is perceived by LecRK-I.9 to trigger plant defence [94]. eATP is considered to be a DAMP (damage associated molecular pattern). ATP recognition triggers increase in cytoplasmic Ca^2+^ concentration followed by MAPK activation and induction of defence-related gene expression; (**B**) LecRK-I.9 interacts with RGD-containing extracellular ligands to mediate contacts between cell wall and plasma membrane [80]. Addition of the RGD-containing effector IPI-O from *Phytophthora* alters the CW-PM continuum leading to defence responses [88]; (**C**) Upon infection by *Pseudomonas* pathogens, LecRK-I.9 is involved in the control of plant defence genes by positively regulating JA-dependent defence genes and by negatively regulating JA-dependent wounding genes [83]. CW: cell wall. PM: plasma membrane. CYT: cytoplasm.

A special attention has been paid to the L-type LecRLK At5g60300 also named LecRK-I.9 [81] or DORN1 (DOES NOT RESPOND TO NUCLEOTIDES) [94] after its identification in two independent screenings [80,94]. First, LecRK-I.9 was selected in a protein-protein interaction screen using the IPI-O effector, a RGD (Arginine-Glycine-Aspartic acid)-containing protein from the oomycete pathogen *Phytophthora infestans*, as bait. The RGD tripeptide motif is a well-known cell adhesion motif in mammalian cells. In plants, the RGD motif has been shown to disrupt cell wall-plasma membrane (CW-PM) contacts [95], and to reduce defence responses when plants are challenged with fungal pathogens [96]. Consistently, a recombinant IPI-O (IN PLANTA INDUCED-O) protein has been shown to disrupt CW-PM contacts in *A. thaliana* [97] and LecRK-I.9 to mediate CW-PM contacts through protein-protein interactions with RGD-containing proteins as potential ligands (Figure 4) [80]. In a study using *lecrk-I.9* mutants, Bouwmeester et al. suggest that the proteins maintaining CW-PM contacts also function in plant defence [88]. Second, LecRK-I.9 was selected in a forward genetic screen for *A. thaliana* mutants impaired in their ability to respond ATP treatment as revealed by cytoplasmic calcium influx [94]. A recombinant extracellular domain of LecRK-I.9 exhibits ATP binding with high affinity and ectopic expression of *LecRK-I.9* increases the plant response to wounding. Notably, ATP and wounding induce a common set of genes. It is assumed that ATP is released during physical damage of cells as a danger signal which is then recognized by LecRK-I.9 (Figure 4) and extracellular ATP is thus proposed to be considered as a Damage-Associated Molecular Pattern (DAMP) [98]. Finally, LecRK-I.9 is located at the plasma membrane as shown by the localization of LecRK-I.9-GFP fusion proteins [88].

Little is known about the signalling pathways and the molecular mechanisms underlying L-type LecRLK-dependent responses. With extracellular ATP as a signal, LecRK-I.9 is required for cytoplasmic Ca^2+^ influx which is the rationale of the selection screen, but also mitogen-activated protein kinase (MPK) activation and gene expression [94] (Figure 4). In particular, *lecrk-I.9* mutants failed to trigger phosphorylation of MPK3 and MPK6. The latter is an important part of the jasmonic acid (JA) signalling pathway and is believed to convert distinct signals (JA, pathogen, cold/salt stress) into different sets of responses in *A. thaliana* [99]. Moreover, upon infection by *Pseudomonas* pathogens, LecRK-I.9 is described as a component of the JA signalling pathway by positively regulating JA-dependent defence genes and by negatively regulating JA-dependent wounding genes at the transcriptional level [83].

The potential ligands for LecRK-I.9, i.e., ATP or/and RGD-containing peptides, are far from what could be expected for a lectin domain facing the cell wall polysaccharides and the *N-* and *O-*glycans of glycoproteins. Modelling of the LecRK-I.9 lectin domain confirms the β-sandwich fold of legume lectins, the conserved residues involved in the Ca^2+^ and Mn^2+^ binding, and the potential carbohydrate-binding site [18,80,100]. The ASYY sequence which is supposed to bind the RGD motif of IPI-O is located in an exposed loop connecting strands β8 and β9, in a position allowing ligand recognition [80]. The latter loop is only found in the *Brassicaceae* family [100]. Docking experiments with ATP show that the ATP binding site has the same location as that of carbohydrates, but the amino acid residues predicted to interact are different [100]. In most cases, the carbohydrate-binding activity of L-type LecRLKs is not demonstrated, except in poplar, where such an activity is detected with α-l-rhamnose as a ligand [101]. The *A. thaliana* L-type LecRLKs are suspected to be devoid of any significant monosaccharide-binding activity. Indeed, the invariant Asp81 key residue responsible for the sugar recognition by canonical legume lectins is replaced by a His residue [18,102]. However, one cannot exclude that the lectin domain of L-type LecRLKs accommodates complex carbohydrates or recruit another lectin domain to restore a functional homodimer. Unfortunately, attempts to crystallize the LecRK-I.9 lectin domain were unsuccessful [103].

The recent progresses in the identification of L-type LecRLKs as critical players during plant-pathogen interactions push forward a common role for this protein family in plant innate immunity [104,105]. In the same way, data about the function of L-type LecPs and LecRLPs are scarce, but suggest that these proteins are involved in the defence response [106,107,108]. Noteworthy, in *M. sativa* plants antisense inhibition of LecPs results in severe abnormalities in embryogenesis and both vegetative and reproductive development are impaired [109]. The identification of the ligands and downstream partners modulating L-type lectin-dependent responses may reveal new functions for this family.

## 5. LysM Receptor-Like Kinases

The Lysin Motif (LysM) consists of about 45 amino acid residues and takes its name from bacterial autolysins which are involved in the remodelling of peptidoglycan [110,111]. As shown in Figure 1, LysM is widely distributed in all kingdoms except in *Archaea* [112]. In plants, it was first identified in the two model legumes *M. truncatula* and *Lotus japonicus* (*L. japonicus*) in the extracellular moiety of the LysM-RLKs identified as putative receptors of Nod factors, the lipo-chitooligosaccharidic (LCO) signalling molecules responsible for establishment of the Rhizobia-legume symbiosis [113,114,115]. Since this discovery, several LysM-containing proteins have been identified from different plants as key players for chitin and peptidoglycan perception (for reviews, see [116,117]), which is not surprising when considering the ability of LysMs to recognize GlcNAc-containing compounds [118]. More surprisingly, a LysM-RLK from *L. jJaponicus* has been recently reported as a receptor able to bind rhizobial exopolysaccharides [119].

In *A. thaliana*, genetic and biochemical approaches have provided evidence for the involvement of AtCERK1/LysM RLK1 [120,121,122,123], AtLYK4 [124] and AtLYK5 [125] in plant innate immunity triggered by chitin and long chain chitin oligomers. However, the molecular mechanisms related to the functioning of these LysM-RLKs are far from being elucidated. The crystal structure of the ectodomain of AtCERK1, solved in complex with a chitin pentamer (GlcNAc)_5_, provides valuable information [123]. It reveals that all three LysMs display the characteristic βααβ structure originally described for the bacterial LysM [112]. Only the LysM2 has been found to interact with the ligand which is anchored in a groove made by the two loops between α1 and β1 on the one hand and α2 and β2 on the other hand. Since LysM2 is able to accommodate only tetra-pentameric chitin oligomers whereas longer chain chitin oligomers are necessary to activate plant immunity [106], a ligand-induced dimerisation mechanism is proposed for the functioning of AtCERK1 [123]. Moreover, AtLYK5 has been identified as an additional chitin receptor component since *Atlyk5* mutants are affected in chitin responses. However, a complete loss of chitin responsiveness, like for *Atcerk1* mutants, has only been observed for *Atlyk4*/*Atlyk5* double mutants*,* suggesting redundancy between *AtLYK5* and *AtLYK4* [125]. AtLYK5 and AtLYK4, like AtCERK1, are chitin binding proteins [122], but unlike AtCERK1, they have an inactive kinase domain. Since the extracellular domain of AtLYK5 exhibits a higher affinity for (GlcNAc)_8_ than that of AtCERK1 [125], the current working model proposes a multi-component receptor complex where the binding of chitin oligomers to AtLYK5 or AtLYK4, induces heteromerisation with AtCERK1 and the subsequent activation of its kinase domain that triggers downstream signalling [125]. In addition to AtCERK1-mediated plant immune response to chitin, another pathway involves the LecP AtLYM2 and possibly an as yet unidentified LysM-RLK, that contributes to defence against fungal pathogens by restricting the molecular flux via plasmodesmata [126].

Besides AtLYM2, two others GPI-anchored proteins, AtLYM1 and AtLYM3 (see Figure 3), physically interact with peptidoglycan but not chitin. *Atlym1* and *Atlym3* mutants exhibit the same defects in peptidoglycan sensitivity and show an enhanced susceptibility to bacterial infection. Interestingly, *Atcerk1* mutants have the same phenotypes as *Atlym1* and *Atlym3* mutants, suggesting that AtCERK1, AtLYM1 and AtLYM3 participate in a peptidoglycan receptor complex [127]. Therefore, AtCERK1 plays a central role as co-receptor for the perception of chitin and peptidoglycan, two important Pathogen-Associated Molecular Patterns (PAMPs). 

In rice, the best characterized chitin receptor complex associates OsCERK1 to OsCEBiP. OsCEBiP, the ortholog of AtLYM2, is the major chitin binding protein in rice exhibiting a high affinity for chitin oligomers [128,129]. The molecular mechanisms leading to the formation and activation of the OsCEBiP/OsCERK1 receptor complex remain hypothetical since the homo-oligomerisation of OsCEBiP upon (GlcNAc)_8_ binding and its interaction with OsCERK1 to activate downstream signalling have not yet been fully elucidated [128,130,131]. In addition, *OsLYP4* (Os09g27890) and *OsLYP6* (Os06g10660), the homologues of *AtLYM1* and *AtLYM3*, exert a dual function in rice immunity by participating in both chitin and peptidoglycan perception [132].

OsCERK1 also intervenes in the symbiosis with arbuscular mycorrhizal fungi [133]. Interestingly, LjNFR1 and MtLYK3, its orthologs in legumes which are required for the establishment of the rhizobial symbiosis, also participate in the mycorrhizal symbiosis [133]. The relationships between these two endosymbioses and basal immunity become more and more documented [134]. For example, MtNFP and LjNFR5, the two kinase-dead LysM-RLKs that are essential for perception of Nod factors and establishment of the rhizobial symbiosis, provoke a hypersensitive reaction when they are co-expressed in *Nicotiana benthamiana* leaves with their respective symbiotic partners MtLYK3 and LjNFR1 [135,136]. MtNFP also controls the susceptibility of *M. truncatula* to pathogen attacks [137]. Furthermore, a possible additional role of Nod factors is the suppression of PAMP-triggered immunity to allow the establishment of symbiosis [138]. Such suppression is also observed in *Arabidopsis* and involves AtLYK3, and this questions the biological significance of Nod factor perception by a species that does not form any root endosymbioses [139]. Future work is needed to understand the molecular basis of the dual function of some LysM-RLKs in symbiosis and immunity and also its origin with respect to the evolution of land plants in order to better understand how plants discriminate between friends and foes.

The establishment of root symbioses both with fungi and bacteria requires in addition to LysM-RLKs that perceive the symbiotic signals, the LecRLK called SYMRK in *L. japonicus* [140] and DMI2/NORK in *Medicago* [141]. These LecRLKs comprise in their extracellular moieties three LRR and a malectin-like domain. In *L. japonicus*, the malectin-like domain is released by a specific cleavage in the absence of symbiotic stimulation [142]. This probably favours the interaction with LjNFR5 and participates in the fine tuning of LjSYMRK within the plasma membrane receptor complex which is required for a successful infection. This association gives a unique example of heterodimerisation of LecRLKs belonging to different lectin families.

## 6. Malectin Receptor-Like Kinases

The malectin CBMs (PF11721) and the structurally related malectin-like domains (PF12819) appear across the animal, bacterial and plant kingdoms (Figure 1). In animals, malectin is a membrane-anchored protein of the endoplasmic reticulum that plays a role in early steps of protein *N*-glycosylation [143]. It is highly conserved in animals and its central part of approximately 190 amino acid residues in length presents a β-sandwich fold. However, the fold differs from the currently known lectins in having α-helices and extensions of the β-sandwich arrangement. Four loops all located at one site of the domain constitute the carbohydrate-binding site with four aromatic residues and one aspartic acid residue mediating the binding. Malectin is able to tolerate a diversity of ligands with di-glucose-high-mannose *N*-glycan as the endogenous ligand [144]. In bacteria, malectin domains are appended to a variety of glycosidase domains (GH2; GH16). In plants, they are appended to kinase domains alone or in association with LRR domains in the extracellular moiety of LecRLKs. The malectin-like domain (PF12819) that is currently found in plant LecRLKs actually includes two divergent malectin domains (PF11721) in tandem. It is then possible that the malectin domains have different functions across the kingdoms.

In *A. thaliana*, malectin-containing LecRLKs fall into two sub-families, namely CrRLK1L and LRR-RLKI (see Section 2, Figure 3). Only few members of the LRR-RLKI subfamily have retained attention. Among them, SIRK1 (SENESCENCE-INDUCED RECEPTOR KINASE 1 encoded by *At2g19190*) is associated with both senescence- and defence-related processes [145]. IOS1 (IMPAIRED OOMYCETE SUSCEPTIBILITY 1 encoded by *At1g51800*) is required for successful infection by biotrophic filamentous oomycete and fungal pathogens [146]. *ios1* mutants are hypersensitive to ABA suggesting that IOS1 participates in the pathogen-mediated outcome by attenuating ABA signalling [143]. Recently, IOS1 has been described to prime pattern-triggered immunity activation [147]. Finally, MEE39 (MATERNAL EFFECT EMBRYO 39 encoded by *At3g46330*) has been identified in a screen using mutants defective in female gametophyte development. In *mee39* mutants, the embryo development is arrested at the one-cell zygote stage [148]. Interestingly, in *M. truncatula*, a malectin-containing LecRLK associates to LysM-LecRLK in a signalling complex for bacterial symbiosis [142] (see Section 5).

The CrRLK1L subfamily comprises 17 LecRLKs and one LecRLP, several members of which have been implicated in the regulation of cell expansion in the context of monitoring the cell wall structure (for review, see [149]). This assumption originates from studies on quickly growing cells during vegetative development, e.g., in dark-grown hypocotyls where cells tremendously elongate from ten µm to one mm-long within a few days. Cell walls should be rigid enough to resist the turgor pressure, but also dynamic to incorporate new cell wall material without rupturing. These opposite processes need to be tightly coordinated in both space and time to maintain growth. THE1 (THESEUS 1) has been identified in a screen to rescue the short hypocotyl phenotype of *procuste* (*prc*) mutants that are deficient in cellulose biosynthesis [150]. Since the double mutant *prc*/*the1* is still deficient in cellulose, the suppressor effect of THE1 argues for its involvement in a sensing mechanism of cell wall perturbations. In the context of damaged cell walls, THE1 inhibits cell growth. However, in leaves and leaf petioles, *THE1* is required for cell elongation together with other members of the malectin LecRLKs subfamily namely, *HERK1/2* (*HERCULES 1/2*) and *FER* (*FERONIA*) [151,152]. The *the1/herk1* double mutant displays shorter cells with an even stronger phenotype in the *the1/herk1/herk2* triple mutant. *fer* mutants show similar cell elongation defect. Then, THE1 function appears to be dependent on the cell wall context. In root hair cells and pollen tubes that are characterized by a polar tip growth, malectin LecRLKs also monitor the cell wall status. *fer* mutants show collapsed or short root hairs that even burst after some growth [153]. The pollen tubes of *anx1*/*anx2* (*anxur 1/anxur 2*) double mutants also burst during growth, thus preventing fertilization. Strikingly, *ANX1/2* are preferentially expressed in pollen tubes and ANX1/2 localize to the plasma membrane of the pollen tube tip [154,155]. In contrast, *FER* is widely expressed throughout plant tissues except in pollen tubes and accumulates in the female gametophyte. In *fer* mutants, pollen tubes fail to rupture and continue to grow invading the embryo sac. Consequently, *fer* mutants embryo sacs remain unfertilized [156,157]. Therefore, when a pollen tube is reaching an ovule, FER in ovules acts in an opposite way to ANX1/2 in pollen tubes. One possible explanation would be that since FER and ANX1/2 share malectin domains in their extracellular moieties, they compete for the same ligands [158]. In this way, malectin-containing LecRLKs exemplify an important cell-to-cell communication process in plants.

The understanding of the signalling networks downstream malectin LecRLKs is increasing rapidly. Cell elongation is tightly associated to cell wall loosening and stiffening, and reactive oxygen species (ROS) are known to mediate the two processes. For instance, hydroxyl radicals are able to break covalent bonds in cell wall polymers, conversely H_2_O_2_ to cross-link cell wall compounds [159,160]. In root hairs, FER has been identified in a protein complex with guanidine nucleotide exchange factors and Rho-GTPases mediating ROS-induced root hair growth [161]. In the female gametophyte, FER is required for the production of hydroxyl radicals causing pollen tube rupture [161]. THE1 and ANX1/2 also regulate ROS production to coordinate the cell wall status and cell expansion [162,163]. CAP1, a tonoplast-localized malectin-LecRLK, functions as a sensor for ammonium signalling in maintaining ROS gradient in root hairs [164]. Other signalling pathways are also concerned by the activity of malectin LecRLKs. In roots, FER binds to a secreted RAFL (RAPID ALKALINISATION FACTOR) peptide that triggers cell wall alkalinization and growth arrest, possibly through the inhibition of plasma membrane ATPase activity [165]. However, the FER domains involved in RAFL binding are not known. FER is also able to recruit a receptor-like cytoplasmic kinase that is phosphorylated by FER in a RALF peptide-dependent manner [166]. Finally, FER is required for the signalling of mechanical forces [167] and the FER/RALF couple is linked to ABA signalling for the control of growth and response to stress [168,169].

Monitoring of cell wall structure and plant innate immunity are closely linked processes [170]. Therefore, malectin LecRLKs may also participate in plant pathogen interactions. For instance, a RALF-mediated inhibition of immunity is FER dependent [171]. Interestingly, *fer* mutants are more resistant to powdery mildew infection [172]. This study suggests similarities between the mechanisms for pollen tube guidance and fungal invasion [173]. A recent study describes TURAN and EVAN that are respectively a UDP-glycosyltransferase and a dolichol kinase involved in protein *N*-glycosylation in the endoplasmic reticulum [174]. The two proteins are required for the interactions between the female and male gametophytes during pollen tube reception. In the ovule, mutations in the two genes phenocopy *fer* mutants. In the pollen, EVAN is required for development and TURAN for pollen tube growth and integrity by affecting the stability of ANX1/2 [24]. Therefore, as for G-type LecRLKs, *N*-glycosylation of malectin LecRLKs is required for proper activity.

## 7. Concluding Remarks

**Figure 5:**
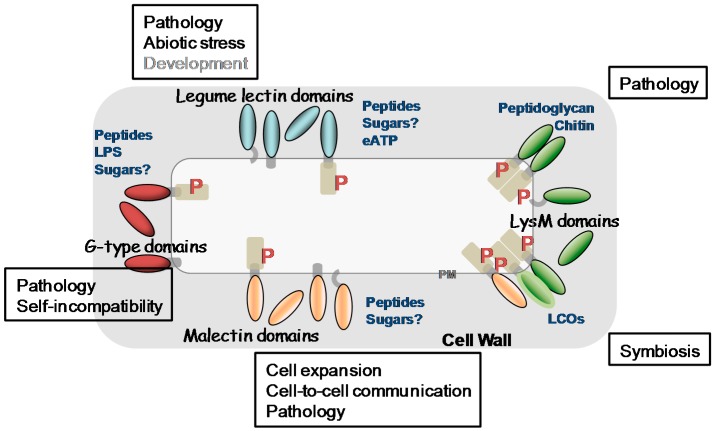
Overview of the main lectin families and lectin receptor kinases at the plant cell surface. G-type (dark red), L-type (blue) lectin domains together with LysM (green) and malectin (orange) CBMs are showed. Their different protein architectures are detailed namely, LecRLKS, LecRLPs and LecPs: some of the latter possess a GPI anchor and thus a potential plasma membrane location. Phosphorylation sites are depicted by a red P. The putative extracellular ligands are indicated in blue. The biological functions in which they have been shown to be involved are in a frame.

In the past decade, our knowledge about plant LecPs, LecRLPs and LecRLKs has greatly increased. These protein families have been shown to play roles during plant development including growth and reproduction, in the perception of biotic and abiotic stresses leading to plant defence reactions as well as in the establishment of symbiosis with microorganisms. Figure 5 summarizes their main functions together with their potential ligands. Detailed diagrams for ligand recognition and signalling mechanisms are available in recent reviews [21,65,138,175]. Several receptor-ligand pairs are now established such as SRK/SCR, LecRK-I.9/RGD-containing peptides or/and ATP, CERK1/chitin oligomers or peptidoglycan, and FER/RAFL. However, regarding the vast expansion of LecRLKs families in plants, only the tip of the iceberg has been revealed. One puzzling result for potential carbohydrate-binding proteins such as LecRLKs lies on the ligands that are not all carbohydrates. Even more, only LysM CBMs give an example of carbohydrate recognition (chitin oligomers/peptidoglycan) by LecPs and LecRLKs. This is unexpected, in particular for malectin LecRLKs involved in monitoring cell wall structure that are expected to find their ligands in the polysaccharidic extracellular matrix [24]. One can consider that the molecular and genetic toolkits used in *A. thaliana* preferentially head for proteins and protein–protein interactions. The emerging plant glycan arrays may reveal a complementary view of LecRLKs ligands [176,177]. Otherwise, it is becoming clear that secreted peptides are important in cell-to-cell communication in plants to coordinate and to integrate cellular functions [178,179] and it is possible that the lectin domains of LecRLKs have evolved towards peptide recognition. In support of this hypothesis are (i) the divergence between the lectin domains of LecRLKs and canonical lectins, (ii) the principle that fold does not necessarily imply function: for instance, many members of the broader family of C-type animal lectins lack sugar-binding activity [10].

Beyond cell signalling, animal lectins also function in the assembly and organization of cell-surface glycoproteins. Galectins, a family of β-galactoside binding proteins, are multivalent and able to crosslink glycoconjugates to form a dynamic lattice [180]. In this way, galectins regulate the functionality of membrane receptors and signalling in many processes. For instance, once bound to *N*-glycans, galectin-3 monomers are able to pentamerize through their N-terminal domains: by recruiting other receptors, the complexes form tubular membrane invaginations in a mechanism of endocytosis [181]. Such mechanisms are not yet described in plants. However, plant endosomal routes also control signalling even if the molecular mechanisms are not very well described and seems to differ depending on the receptors [182]. Finally, organizing the plant cell-surface has been shown critical in cell differentiation and reproduction, e.g., rupture of the pollen tube cell wall upon contact with the embryo sac leading to the release of sperm cells [154,183]. We anticipate that further studies on cell-surface lectins and lectin receptor kinases will impact many aspects of plant biology.

## Supplementary Materials

Supplementary materials can be found at www.mdpi.com/1422-0067/18/6/1164/s1.
